# Genome Wide Identification, Phylogeny and Expression of Zinc Transporter Genes in Common Carp

**DOI:** 10.1371/journal.pone.0116043

**Published:** 2014-12-31

**Authors:** Yanliang Jiang, Songhao Zhang, Shuaisheng Feng, Jinsheng Sun, Peng Xu

**Affiliations:** 1 CAFS Key Laboratory of Aquatic Genomics and Beijing Key Laboratory of Fishery Biotechnology, Centre for Applied Aquatic Genomics, Chinese Academy of Fishery Sciences, Beijing, 100141, China; 2 College of Life Sciences, Tianjin Normal University, Tianjin, 300387, China; University of Florida, United States of America

## Abstract

**Background:**

Zinc is an essential trace element in organisms, which serves as a cofactor for hundreds of enzymes that are involved in many pivotal biological processes including growth, development, reproduction and immunity. Therefore, the homeostasis of zinc in the cell is fundamental. The zinc transporter gene family is a large gene family that encodes proteins which regulate the movement of zinc across cellular and intracellular membranes. However, studies on teleost zinc transporters are mainly limited to model species.

**Methodology/Principal Findings:**

We identified a set of 37 zinc transporters in common carp genome, including 17 from SLC30 family (ZnT), and 20 from SLC39 family (ZIP). Phylogenetic and syntenic analysis revealed that most of the zinc transporters are highly conserved, though recent gene duplication and gene losses do exist. Through examining the copy number of zinc transporter genes across several vertebrate genomes, thirteen zinc transporters in common carp are found to have undergone the gene duplications, including SLC30A1, SLC30A2, SLC30A5, SLC30A7, SLC30A9, SLC30A10, SLC39A1, SLC39A3, SLC39A4, SLC39A5, SLC39A6, SLC39A7 and SLC39A9. The expression patterns of all zinc transporters were established in various tissues, including blood, brain, gill, heart, intestine, liver, muscle, skin, spleen and kidney, and showed that most of the zinc transporters were ubiquitously expressed, indicating the critical role of zinc transporters in common carp.

**Conclusions:**

To some extent, examination of gene families with detailed phylogenetic or orthology analysis could verify the authenticity and accuracy of assembly and annotation of the recently published common carp whole genome sequences. The gene families are also considered as a unique source for evolutionary studies. Moreover, the whole set of common carp zinc transporters provides an important genomic resource for future biochemical, toxicological and physiological studies of zinc in teleost.

## Introduction

Zinc (Zn^2+^) is an essential trace element that plays important role in cells. This mineral serves as a cofactor for hundreds of enzymes that are involved in many pivotal biological processes including growth, development, reproduction and immunity [1]. Deficiency or excessive accumulation of zinc causes a wide range of physiological defects including impairments in growth, development, neural function and immune system [2]. Therefore, the homeostasis of Zn^2+^ in the cell is fundamental for the survival of living organisms. Zinc transporters are transmembrane proteins that regulate the movement of zinc across cellular and intracellular membranes [3]. There are two families of zinc transporter, SLC30, also referred as ZnT, and SLC39, referred as ZIP, functioning in opposite directions to maintain cellular zinc homeostasis [4]. ZnTs maintain the cytoplasmic zinc balance by exporting zinc out to the extracellular space or by sequestrating cytoplasmic zinc into intracellular compartments such as endoplasmic reticulum, Golgi, mitochondira and vesicle, when cellular zinc levels are elevated [4]. In contrast, ZIPs can increase the zinc concentration when cellular zinc level is low, by promoting the zinc into the cytoplasm from the extracellular space of the cell or from the intracellular storage compartment [5].

The first zinc transporter was cloned and discovered in human in 1995, the ZnT1 [6]. Since then, rapid progress have been made on zinc transporter researches including discovering the new members, understanding the physiological roles, and identification of gene expression patterns. In human and mammals, ten members of the ZnT family have been identified. Most ZnT proteins have six transmembrane (TM) domains, with an exception of ZnT5 with 12 or more TM domains [7]. In addition, most ZnTs are predicted to possess cytoplasmic amino and carboxy termini, and have a long histidine-rich loop between the 4^th^ and the 5^th^ TM [7]. The highly conserved domains appear critical for zinc transport [7]. For ZIP family, fourteen members have been reported by far. Slightly different from the topology of ZnTs, there are eight TM domains for most ZIPs with extracellular amino and carboxy termini [7]. The common feature of ZIP proteins is a long loop region between the third and the fourth TM domain, although the length and sequences of this loop region is not well conserved [7].

Comparing with that in mammals, the zinc homeostasis in teleost is more complicated, since teleost fish live in the aquatic environment and is subject to greater pressure than living in the aerial environment. Due to the importance of zinc transporters in the teleost zinc homeostasis, extensive studies have been conducted. For instance, Yan et al. reported that SLC39A7 played critical role in the formation of eye, brain and skeleton during early embryonic development in zebrafish [8]. Feeney et al. mined zinc transporters from three teleost genomes including zebrafish, *fugu* and *Tetraodon*, and concluded the evolutionary conservation of these genes between mammals and teleost [9]. However, studies on teleost zinc transporters have been mainly limited to model species due to the limitation of genomic resource in non-model fish species.

Common carp, *Cyprinus carpio*, one of the most significant aquaculture fish species, is widespread all over the word especially in Europe and Asia. Great efforts have been made in developing genomic resources in recent years. These genomic resources included a large number of ESTs [10], BAC end sequences [11], comprehensive transcriptome obtained by RNA seq [12], [13], single nucleotide polymorphism (SNPs) [14], genetic and physical maps [15], [16]. The common carp whole genome sequences have recently been published [17]. It is known that common carp genome is allotetraploidized genome which had experienced additional round of whole genome duplication (WGD) comparing with most other teleost. Therefore, the complexity of the tetraploidized genome and gene duplications may cause misidentification in assembly and annotation. Examination of gene families with phylogenic or orthology analysis would verify the whole genome sequences assembly and annotation [18]. In this study, by utilizing all available common carp genomic resources, we identified 37 zinc transporter genes across the genome. Further phylogenetic and syntenic analysis confirmed the annotation. Moreover, we examined the tissue distribution of zinc transporter genes in common carp. The expression patterns of each gene, together with the information of orthologs identified from other species, were used for the inference of the potential function of zinc transporter genes in common carp. Our study on examining gene families in common carp not only supported the accuracy of the common carp whole genome sequences assembly and annotation, but also provided valuable genomic resources for the future evolutionary, biochemical, toxicological, and physiological studies on common carp and other teleost.

## Results and Discussion

### Identification of Zinc Transporters in Common Carp

The mammalian SLC30 transporters belong to a large superfamily of transporters called cation diffusion facilitator that includes zinc transporters with similar topology among bacteria, fungi, nematodes, insects, plants, and mammals [19]. Through utilizing all available genomic resources, here, we identified a total of 37 zinc transporter genes in common carp genome, including 17 SLC30 members and 20 SLC39 members. All coding sequences of zinc transporter genes were deposited to DDBJ database with continuous accession number of AB987974-AB988010 (Table 1). Detailed information of their corresponding genomic sequences, coding sequences, number of exons are summarized in Table 1.

**Table 1:** Summary of SLC30 and SLC39 families in common carp genome.

Gene name	Genomic length(bp)	CDS (na)	CDS(aa)	CDS status	No. of exons	Accession
**SLC30A1a-1**	2,353	912	303	Partial	2	AB987974
**SLC30A1a-2**	1,413	924	307	Partial	2	AB987975
**SLC30A1b-1**	6,026	1,449	482	Complete	5	AB987976
**SLC30A1b-2**	3,185	816	271	Partial	3	AB987977
**SLC30A2-1**	4,891	1,062	353	Complete	8	AB987978
**SLC30A2-2**	3,274	717	238	Partial	5	AB987979
**SLC30A4**	20,335	1,281	426	Complete	9	AB987980
**SLC30A5-1**	2,747	2,319	772	Complete	4	AB987981
**SLC30A5-2**	11,219	1,737	578	Complete	13	AB987982
**SLC30A6**	31,909	1,683	560	Complete	9	AB987983
**SLC30A7-1**	6,948	1,167	388	Complete	5	AB987984
**SLC30A7-2**	2,827	954	317	Partial	4	AB987985
**SLC30A8**	11,702	2,028	675	Complete	16	AB987986
**SLC30A9-1**	7,111	1,722	573	Complete	13	AB987987
**SLC30A9-2**	11,437	2,325	774	Complete	20	AB987988
**SLC30A10-1**	9,988	1,242	413	Partial	8	AB987989
**SLC30A10-2**	1,738	1,074	357	Partial	3	AB987990
**SLC39A1-1**	4,669	867	288	Complete	4	AB987991
**SLC39A1-2**	3,836	894	297	Complete	4	AB987992
**SLC39A3-1**	741	879	292	Complete	3	AB987993
**SLC39A3-2**	1,740	945	314	Complete	3	AB987994
**SLC39A4-1**	12,485	1,665	554	Complete	8	AB987995
**SLC39A4-2**	10,644	1,986	661	Complete	15	AB987996
**SLC39A4-3**	14,255	1,692	563	Partial	8	AB987997
**SLC39A5-1**	6,194	1,998	665	Complete	15	AB987998
**SLC39A5-2**	5,684	1,737	578	Complete	12	AB987999
**SLC39A6-1**	4335	1,917	638	Partial	13	AB988000
**SLC39A6-2**	6516	2,067	688	Complete	10	AB988001
**SLC39A7-1**	2,178	582	193	Partial	5	AB988002
**SLC39A7-2**	4,473	831	276	Partial	6	AB988003
**SLC39A8**	6,396	1,329	442	Complete	8	AB988004
**SLC39A9-1**	6,527	927	308	Complete	7	AB988005
**SLC39A9-2**	4,333	735	284	Complete	6	AB988006
**SLC39A10**	74,625	2,493	830	Complete	9	AB988007
**SLC39A11**	157,166	855	284	Partial	4	AB988008
**SLC39A13**	11,141	1,134	377	Complete	9	AB988009
**SLC39A14**	17,254	1,470	489	Complete	4	AB988010

For SLC30 family, a total of 17 members were identified in the common carp genome, including SLC30A1a-1, SLC30A1a-2, SLC30A1b-1, SLC30A1b-2, SLC30A2-1, SLC30A2-2, SLC30A4, SLC30A5-1, SLC30A5-2, SLC30A6, SLC30A7-1, SLC30A7-2, SLC30A8, SLC30A9-1, SLC30A9-2, SLC30A10-1, SLC30A10-2 (Table 1). Full length coding sequences were obtained for 10 of the 17 SLC30 family members. For SLC39 family, 20 members were identified in common carp, including SLC39A1-1, SLC39A1-2, SLC39A3-1, SLC39A3-2, SLC39A4-1, SLC39A4-2, SLC39A4-3, SLC39A5-1, SLC39A5-2, SLC39A6-1, SLC39A6-2, SLC39A7-1, SLC39A7-2, SLC39A8, SLC39A9-1, SLC39A9-2, SLC39A10, SLC39A11, SLC39A13, and SLC39A14. Full length coding sequences were obtained for 15 members of all 20 SLC39 members (Table 1). The genomic structure of each gene member of SLC30 and SLC39 are shown in S1 Fig. and S2 Fig.

**Figure 1:**
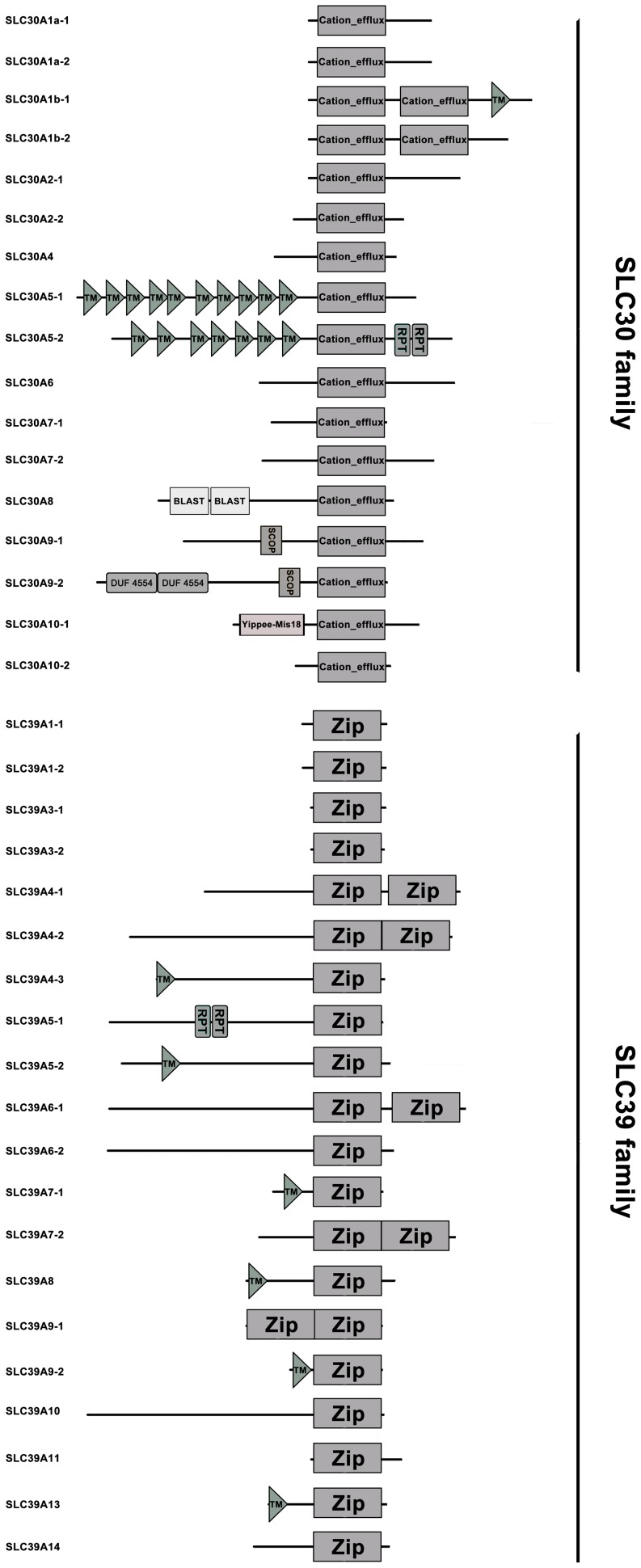
Schematic representation of the domain architecture of zinc transporters in common carp. TM represents the transmembrane region; RPT represents the internal repeat 1; BLAST represents the domain present in proteins and involved in regulation of nuclear pre-mRNA; SCOP represents the putative copper-binding protein; DUF4554 represents the domain of unknown function 4554; and Yippee-Mis18 represents the Yippee zinc-binding/DNA-binding/Mis18.

As shown in Fig. 1, all SLC30s possess one Cation_efflux domain except for SLC30A1b-2 and SLC30A2-2 with two Cation_efflux domains. Cation_efflux domain functions as efflux pumps that remove cations such as zinc ions from cells, and it contains various number of transmembrane domain (TM). ZnT5 (SLC30A5-1 and SLC30A5-2) is the only exception which contains additional TM at the N-terminal end of the protein. This finding is consistent with the structure of mammalian ZnTs [4], indicating the high conservation of the ZnTs. All SLC39s possess at least one Zip domain, of which SLC39A4-1, SLC39A4-2, SLC39A6-1, SLC39A7-2 and SLC39A9-1 possess two Zip domains (Fig. 1).

### Phylogenetic Analysis of SLC30 Transporters

**Figure 2:**
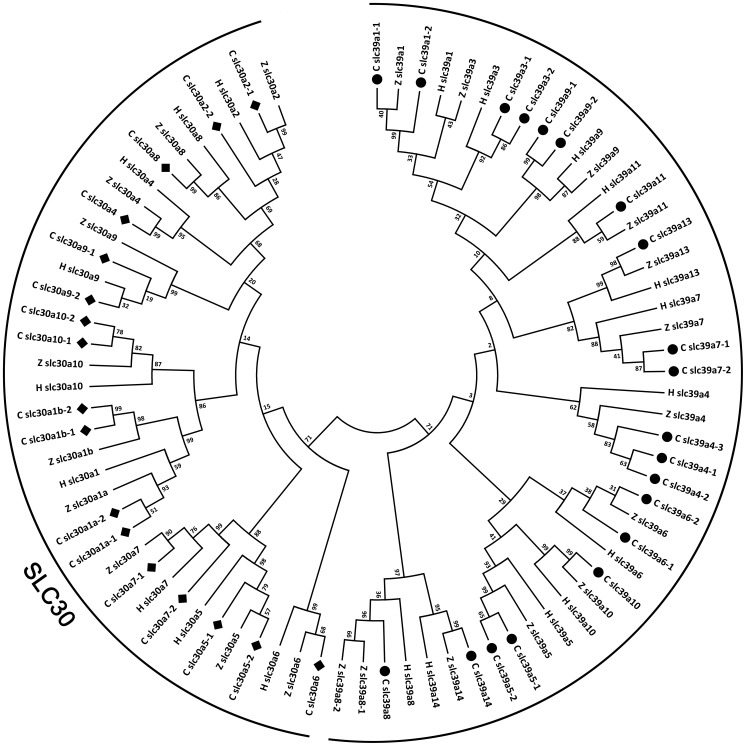
The common carp zinc transporter gene family. Numbers around the nodes correspond to bootstrap support values. Abbreviations: H: *Homo sapiens*; Z: *Danio rerio* and C: *Cyprinus carpio*. The black dots/diamonds indicate common carp genes.

The phylogenetic analysis can be used to support the gene annotation, especially for non-model species [18]. Reference zinc transporter genes from representative vertebrate model species were used for phylogenetic analysis, including *Homo sapiens* (human), *Mus musculus* (mouse), *Gallus gallus* (chicken), *Anolis carolinensis* (lizard), *Xenopus* (frog), *Oryzias latipes* (medaka), and *Danio rerio* (zebrafish). The phylogenetic analysis results showed that each of common carp zinc transporter genes clustered with its respective counterpart from other species (Fig. 2), indicating all genes in zinc transporter gene family are highly conserved.

**Figure 3:**
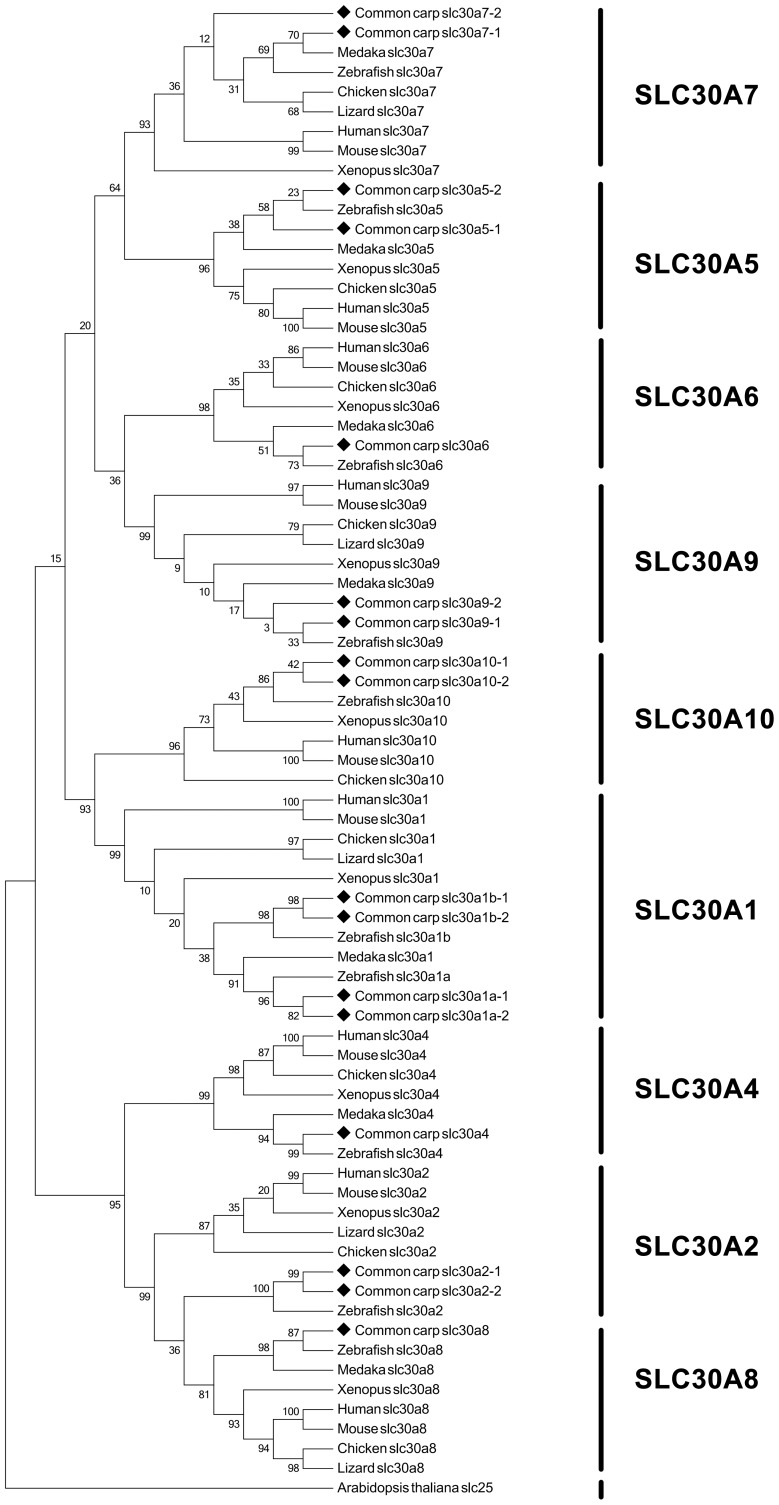
Phylogenetic tree of SLC30 family transporters. The phylogenetic tree was constructed using maximum likelihood algorithm under the JTT+I+G model of amino acid substitution as described in detail in Materials and Methods section. Numbers around the nodes correspond to bootstrap support values in percentages.

For SLC30 family, as revealed by phylogenetic tree, four major clades were formed, with SLC30A7 and SLC30A5 in a clade, SLC30A6 and SLC30A9 in a clade, SLC30A10 and SLC30A1 in a clade, SLC30A4, SLC30A2 and SLC30A8 in a clade, respective (Fig. 3). We speculated that these genes falling into one clade may be derived from a very ancient lineage of vertebrate. Similar relationships of these genes have been reported in mammalian SLC30 genes, which therefore are divided into four subfamilys [4].

**Figure 4:**
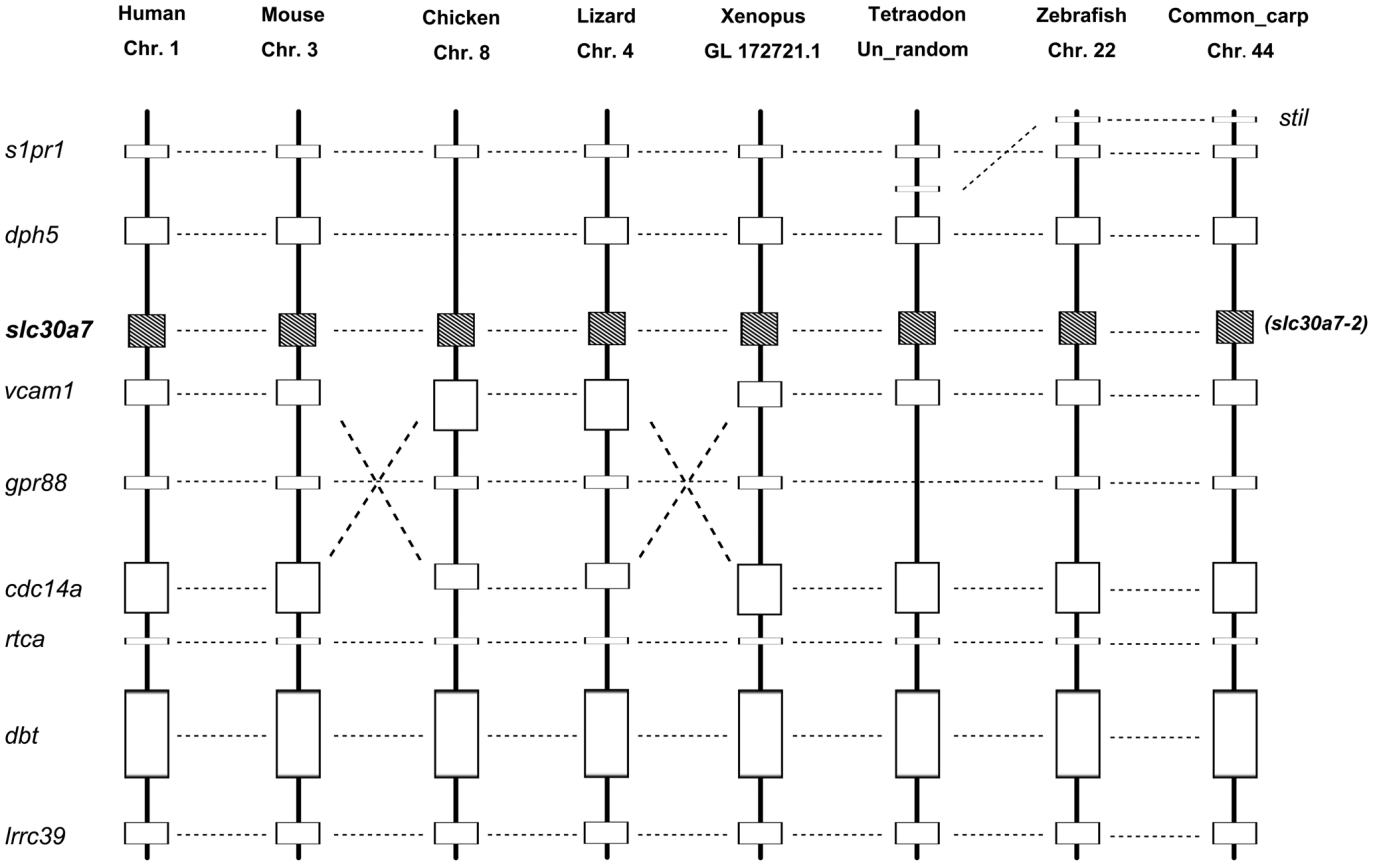
Analysis of conserved synteny blocks harboring SLC30A7 gene in several vertebrates. Horizontal lines denote orthologous relationships. Abbreviations: s1pr1: Sphingosine 1-phosphate receptor 1; dph5: Diphthine synthase 5; slc30a7: Solute-carrier gene family 30, member 7; vcam1: Vascular cell adhesion protein 1; gpr88: Probable G-protein coupled receptor 88; cdc14a: Cell division cycle 14 homolog A; rtca: RNA 3′-terminal phosphate cyclase; dbt: Dihydrolipoamide branched chain transacylase; lrrc39: Leucine rich repeat containing 39.

However, the common carp SLC30A7-1 and its homologous-copy SLC30A7-2 didn't fall into a distinct clade. As shown in Fig. 3, SLC30A7-1 is placed into a subclade with the orthologous from zebrafish, medaka, chicken and lizard, but SLC30A7-2 is placed out of the subclade. Therefore, phylogenetic analysis alone did not provide a solid support for the annotation of the common carp SLC30A7-2. We therefore conducted syntenic analysis of this gene. As shown in Fig. 4, the annotation of common carp SLC30A7-2 is apparently supported by syntenic analysis by comparing the genomic region containing SLC30A7-2 in human, mouse, chicken, lizard, *Xenopus*, *Tetraodon*, zebrafish and common carp. The gene order is well conserved, even with an exception of an inversion of VCAM1 and CDC14A between chicken and lizard.

**Figure 5:**
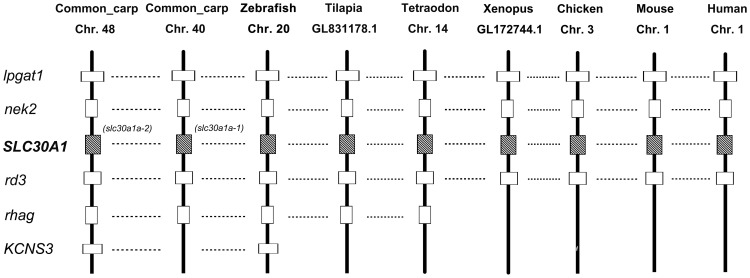
Analysis of conserved synteny blocks harboring SLC30A1 gene in several vertebrates. Horizontal lines denote orthologous relationships. Abbreviations: lpgat1: Lysophosphatidylglycerol acyltransferase 1; nek2: NIMA (never in mitosis gene a)-related kinase 2; slc30a1: Solute-carrier gene family 30, member 1; rd3: Retinal degeneration 3; rhag: Rhesus blood group-associated glycoprotein; kcns3: Potassium voltage-gated channel, delayed-rectifier, subfamily S, member 3.

To further confirm the annotation of the remaining SLC30 genes, another gene member SLC30A1 was selected for the syntenic analysis as well, which has four orthologous copies in common carp. As shown in Fig. 5, it is apparent that the annotation of the common carp SLC30A1 was supported by the conserved syntenies. In the genomic neighborhood containing the SLC30A1 gene, the gene order was well conserved, with lpgat1, nek2 on the one side of the SLC30A1 gene, and rd3 gene on the other side of the SLC30A1 gene in the genomes of human, mouse, chicken, *Xenopus*, *Tetraodon*, tilapia, zebrafish and common carp (Fig. 5). Apparently, SLC30A1a-1 located in common carp chromosome 40 and SLC30A1a-2 located in chromosome 48 are the result of gene duplication in carp genome, based on their highly conserved syntenic regions (Fig. 5).

### Phylogenetic Analysis of SLC39 Transporters

**Figure 6:**
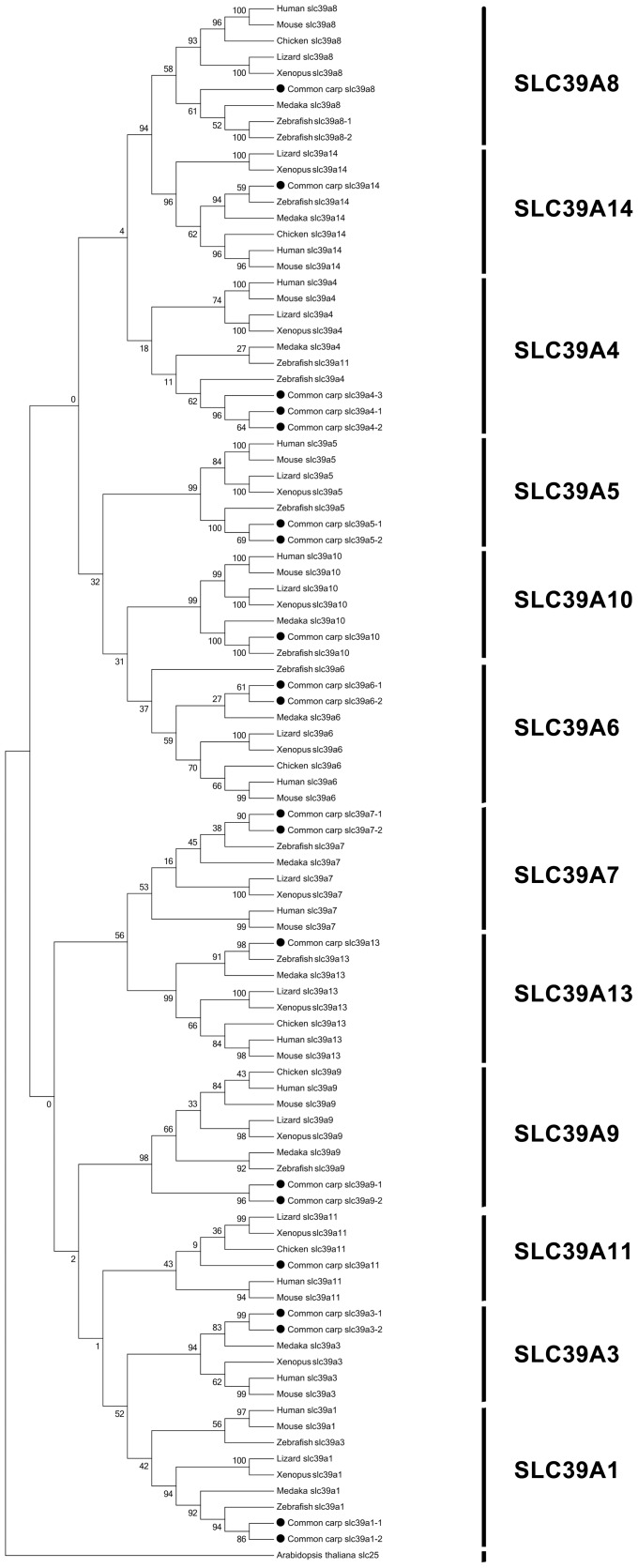
Phylogenetic tree of SLC39 family transporters. The phylogenetic tree was obtained as in Fig. 3. Numbers around the nodes correspond to bootstrap support values.

Overall, phylogenetic analysis well supported the annotations of common carp SLC39 genes. All the common carp SLC39 genes fell into their respective clades with orthologous genes from other species (Fig. 6). SLC39A8 had two copies in zebrafish genome, but there was only one copy in common carp. So we could conjecture that the common carp SLC30A8 experienced potential gene decrease during the evolution. SLC39A4 is the only one that has 3 copies in SLC39 family. Phylogenetic tree showed that SLC39A4-1 and SLC39A4-2 grouped together first and then formed one clade with common carp SLC39A4-3, indicating that the duplication of SLC39A4-1 and SLC39A4-2 might occurred after the appearing of the SLC39A4-3. Interestingly, zebrafish SLC39A3 fell into the clade of SLC39A1s. We further investigated the sequences of zebrafish SLC39A3, and found that the similarity of zebrafish SLC39A3 with human SLC39A3 was only 23% while the similarity of zebrafish SLC39A3 with human SLC39A1 was 35%, suggesting that zebrafish SLC39A3 gene highly likely is another copy of SLC39A1. SLC30A5, SLC30A6 and SLC30A10 fell into a subclade, which is consistent with other mammalian studies [20], [21]. However, intriguingly in our results, SLC39A10 was firstly grouped with SLC39A5 when SLC39 orthologous from only three species were used (Fig. 2), while SLC39A10 was firstly grouped with SLC30A6 when more SLC39 orthologous were added (Fig. 6). The latter is in agreement with previous data reported in mammalian systems [20], [21]. This might be caused by two reasons: firstly, the sequence similarities of those three paralogous are very high, and the protein structures are highly conserved [21]. They are phylogenetically close to each other, therefore make it relatively difficult to tell which two are closer. Secondly, it is acceptable that adding more orthologous for phylogenetic analysis results in slightly different phylogenetic trees. The more orthologous are used, the more reliable the phylogenetic tree would be considered to be.

### Gene Duplications and Losses of Zinc Transporters in Common Carp

Whole-genome duplication (WGD) is one of the major drivers that have shaped the evolutionary history of many vertebrates. Since Ohno suggested that two rounds of large-scale gene duplication had occurred early in vertebrate evolution [22], a number of studies on the comparative analysis of Hox gene clusters provided solid evidences in support of Ohno's hypothesis [23]–[26]. An additional round of duplication, also named teleost-specific (TS) WGD, or the 3R WGD, took place in the common ancestor of all extant teleost. As a result of genome duplication, teleost fish have two paralogous copies for many genes, while only one ortholog is present in tetrapods.

For salmonids and some cyprinids such as common carp and goldfish, additional TS WGD (the 4R WGD) occurred, according to estimations probably between 5.6–11.3 MYA [27]. Analysis of microsatellite loci [28] and comparing common carp linkage map to zebrafish genome [29] provided critical evidence in support of the 4R duplication event in common carp. Followed by whole genome duplication, lineage-specific paralog duplication and loss are frequently observed during evolution. In this study, by examining the copy number of zinc transporter genes in several vertebrate genomes, with focus on common carp and other fish with sequenced genomes, we inferred thirteen members of zinc transporters in common carp have undergone the gene duplications, including SLC30A1, SLC30A2, SLC30A5, SLC30A7, SLC30A9, SLC30A10, SLC39A1, SLC39A3, SLC39A4, SLC39A5, SLC39A6, SLC39A7 and SLC39A9 (Table 2). These genes are highly likely duplicated as a result of the 4R WGD, because double or more copies of respective gene were present in common carp than in its closely-related model fish species zebrafish. In addition to the duplication, lineage-specific gene losses were observed as well. For instance, single copy of SLC30A4, SLC30A6, SLC30A8, SLC39A8, SLC39A10, SLC39A11, SLC39A13 and SLC39A14 were observed in common carp genome suggesting potential gene losses after whole genome duplication (Table 2). SLC39A2 was not found in any fish genome, which appeared as a result of gene loss after the split of teleost fish from tetrapods (Table 2).

**Table 2:** Comparative analysis of zinc transporters of common carp with other species.

	Human	Mouse	Chicken	Xenopus	Medaka	Fugu	Stickleback	Tetraodon	Tilapia	Cod	Coelacanth	Zebrafish	Common_carp
***ZnT***													
**SLC30A1**	1	1	1	1	1	1	1	1	1	1	1	2	4
**SLC30A2**	1	1	1	1	0	0	1	1	0	1	1	1	2
**SLC30A3**	1	1	0	1	1	1	0	0	1	0	1	0	0
**SLC30A4**	1	1	1	1	1	1	1	1	1	1	1	1	1
**SLC30A5**	1	1	1	1	1	1	1	1	1	1	1	1	2
**SLC30A6**	1	1	1	1	1	1	1	1	1	1	1	1	1
**SLC30A7**	1	1	1	1	1	1	1	1	1	1	1	1	2
**SLC30A8**	1	1	1	1	1	1	1	1	1	1	1	1	1
**SLC30A9**	1	1	1	1	1	1	1	1	1	0	1	1	2
**SLC30A10**	1	1	1	1	0	0	0	0	0	0	2	1	2
***ZIP***													
**SLC39A1**	1	1	0	1	1	1	1	1	2	1	2	1	2
**SLC39A2**	1	1	0	1	0	0	0	0	0	0	0	0	0
**SLC39A3**	1	1	1	1	1	0	1	1	1	1	1	1	2
**SLC39A4**	1	1	0	1	1	1	1	1	1	1	1	1	3
**SLC39A5**	1	1	0	1	0	0	0	0	0	0	1	1	2
**SLC39A6**	1	1	1	1	1	1	1	2	1	1	1	1	2
**SLC39A7**	1	1	0	1	1	1	0	1	1	1	1	1	2
**SLC39A8**	1	1	1	1	1	1	1	1	1	1	1	2	1
**SLC39A9**	1	1	1	1	1	1	1	1	1	1	1	1	2
**SLC39A10**	1	1	0	1	1	1	1	1	1	1	1	1	1
**SLC39A11**	1	1	1	1	0	1	1	1	0	1	1	1	1
**SLC39A12**	1	1	1	1	1	1	1	1	1	0	1	0	0
**SLC39A13**	1	1	1	1	1	1	1	1	1	1	1	1	1
**SLC39A14**	1	1	1	1	1	1	1	1	1	1	1	1	1

### Expression Profiling of Zinc Transporter Genes of Common Carp and Potentially Functional Inferences

**Figure 7:**
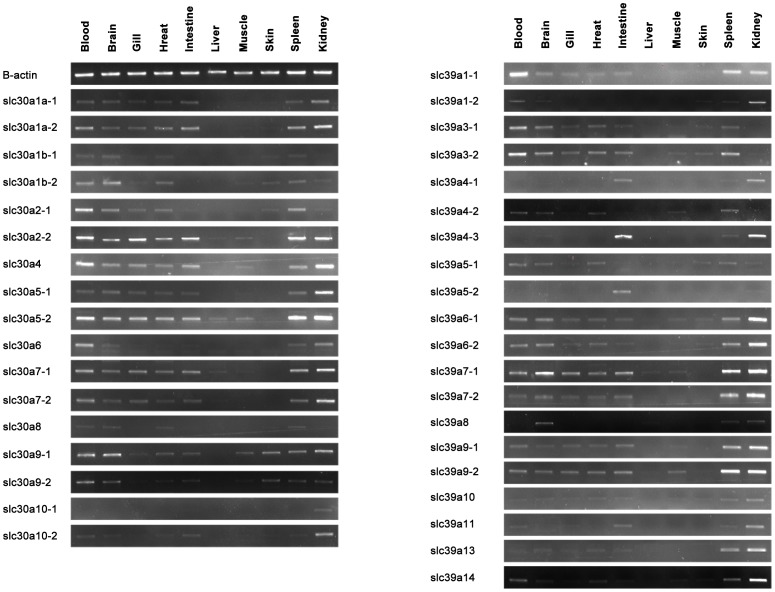
RT-PCR based expression analysis of common carp zinc transporter genes. The amplification of β-actin was used as an internal control. Gene names are indicated on the left of the panel.

Given the unexpected expansion of the zinc transporters gene family in common carp, it was of interest to examine how many of these genes are actually expressed, and to determine the expression pattern of these genes. In order to achieve these objectives, we conducted RT-PCR using gene-specific primers to examine the expression pattern of each gene in various common carp tissues. As shown in Fig. 7, both SLC30 and SLC39 families exhibit unique tissue-specific expression. In general, most of the zinc transporter genes are widely expressed, but with a relatively high expression level in blood, brain, gill, heart, intestine, spleen and kidney (Fig. 7), since these tissues are more likely to be involved zinc acquisition, recycling or transfer.

The zinc transporters play important roles in various physiological processes, with a large portion of which involved in diverse physiological diseases [30], [31]. Extensive functional studies have been performed in mammals, but specific functions in fish are largely unknown. The establishment of gene expression pattern, together with the information of orthologous from model species should provide potential for functional inferences, with the understanding that the function of lineage specific genes can be distinct depending on living environments under different selection pressure [18]. Functional inferences for zinc transporter genes that have undergone duplications or losses in teleost fish are of most interest because they are potentially underlying the adaptations to aquatic environments.

#### SLC30s (ZnTs)

In mammals, the expression of ZnT1 has a wide tissue distribution [32]. Different from mammals with one copy of ZnT1, four copies of ZnT1 were identified in common carp. Of the four copies, only SLC30A1a-2 is highly expressed in most of the tissues. The other three are highly expressed in distinctive tissues. Gene expressed in different cells or tissues may have different functions. Based on the hypothesis of subfunctionalization of WGD-derived duplicates, duplicated genes may be retained with portions of the function of the ancestral gene [33]. SLC30A1 had been demonstrated an essential role in embryonic development, maternal zinc transport to embryos, and in maintenance of cellular zinc homeostasis in SLC30A1 KO mice [34]. Female heterozygous SLC30A1 KO mice are more susceptible to dietary zinc deficiency-induced embryonic development abnormality during pregnancy than the wild type control, suggesting that SLC30A1 is essential in maintaining normal zinc homeostasis in adult mice [4].

As shown in Fig. 7, the expression of SLC30A2, SLC30A4, SLC30A5, SLC30A7 and SLC30A9 have a wide tissue distribution, with slight difference of distribution pattern between the two copies of each gene. SLC30A2 (ZnT2) is believed to participate in apoptosis [35], zinc export in mammary glands [36], and affecting normal growth and development of infants [37], although no animal KO model of ZnT2 has been established yet. In mammals, ZnT2 expressed as a highest level in intestine, kidney [38], which is consistent with our result (Fig. 7), indicating the similar function of ZnT2 in common carp. Mammalian ZnT4 controls zinc deposition in milk [39], and maintains body zinc homeostasis in middle- or late-adulthood [40]. ZnT5 plays important role in development by regulating the secretion of growth hormone [41]. Mice lacking ZnT5 grow poorly and are lean [42]. The null mutation of SLC30A7 in mice results in retarded growth and a lean phenotype. SLC30A7 KO mice are zinc deficient evident by low serum zinc levels and reduced zinc contents in major organs including liver, intestine, kidney, and bone [43].

The expression of SLC30A6, SLC30A8 and SLC30A10 is observed only in a few tissues of common carp (Fig. 7). Differently from human SLC30A6 that was predominantly expressed in brain, liver, and intestine [44], [45], our result showed highly expression of SLC30A6 were observed in blood and kidney in common carp (Fig. 7). Human SLC30A8 mRNA was only detected in the brain and liver tissues [46], while high expression level were detected in blood, brain, heart and spleen in common carp (Fig. 7). Different tissue distribution of these genes indicated their functions are highly likely to be different among different species.

#### SLC39s (ZIPs)

Overall, the expression level and pattern of SLC39s are various in different tissues of common carp (Fig. 7). SLC39A1, SLC39A3, SLC39A5, SLC39A6, SLC39A7 and SLC39A9 are ubiquitously expressed in common carp tissues, while the remaining genes are mainly expressed in only two or three tissues. In mammals, most of the ZIPs are widespread expressed in tissues, including SLC39A1, SLC39A2, SLC39A3, SLC39A6, SLC39A7, SLC39A8, SLC39A13 and SLC39A14, while the remaining genes are expressed only in specific cells or tissues [20]. SLC39A1, SLC39A2, SLC39A3 and SLC39A8 were reported to be zinc uptake transporters, and associated with growth hormone level in the body [47]–[50]. SLC39A4 is involved in zinc absorption and significantly up-regulated during zinc deficiency [51]. SLC39A5 is thought to play unique role in polarized cells by sensing zinc status [52], and regulate intestinal zinc excretion to protect against zinc-induced acute pancreatitis [53]. SLC39A7 is involved in the formation of eye, brain and skeleton during early embryonic development [8]. Other SLC39s such as SLC39A6, SLC39A8, SLC39A10 and SLC39A14 play critical roles in immune system [54]–[58].

## Conclusions

A total of 37 zinc transporters were identified from common carp genome. Phylogenetic analysis, along with syntenic analysis if necessary, allowed annotation of these transporters. Our result showed that the majority of zinc transporters were well conserved through evolution. Clear orthologous relationships were established for the majority of zinc transporter genes, enabling the possibility for functional inference of the common carp transporters. Most of the zinc transporter genes were ubiquitously expressed in common carp, but highly expressed in the issues that are more likely to be involved zinc acquisition, recycling or transfer, indicating the critical roles of this gene family in maintaining zinc homeostasis. However, detailed functions of each gene need further studies. The whole set of zinc transporters provided essential genomic resources for future biochemical, toxicological, physiological and evolutionary studies in common carp and other teleost.

## Materials and Methods

### Ethics statement

This study was approved by the Animal Care and Use committee of the Centre for Applied Aquatic Genomics at the Chinese Academy of Fishery Sciences.

### Identification of SLC30 and SLC39 Transporter Genes and Homologs

All available SLC30 and SLC39 transporters from zebrafish and human were downloaded from Ensembl (http://www.ensembl.org) and used as queries to search against all available common carp genomic resource including whole genome sequences, transcriptome sequences, cDNAs by BLAST searches to acquire the candidate genes with an E-value cutoff of 1e-5. Then reciprocal BLAST searches were conducted by using the candidate common carp zinc transporter genes as queries to verify the veracity of candidate genes. Additionally, the coding sequences were confirmed by BLAST searches against NCBI non-redundant protein sequence database (nr). The simple modular architecture research tool (SMART) was used to predict the conserved domains based on sequence homology and further confirmed by conserved domain prediction from BLAST. The full-length amino acid sequences as well as the partial sequences coding for the conserved domains were used in the phylogenetic analysis. The SLC30 and SLC39 proteins from other organisms were retrieved from the Ensembl genome database (Release 75) for phylogenetic analysis with exclusion of partial sequences.

### Phylogenetic and Orthology Analysis

For the sake of annotating the SLC30 and SLC39 transporters, phylogenetic analysis was conducted with reference SLC30 and SLC39 proteins from zebrafish, human, and other representative vertebrate species. The gene names and accession numbers of the reference SLC30s and SLC39s used in this study are shown in S1 Table. For nomenclatures of the common carp SLC30 and SLC39, whenever possible we followed those of zebrafish because zebrafish is the most closely related model species to common carp. Multiple protein sequences were aligned by ClustalW with default parameters. We performed maximum likelihood analysis in MEGA6 with bootstrap test of 1000 replicates. The best-fit model was the JTT+I+G model which uses a Jones-Taylor-Thornton (JTT) matrix and incorporates a proportion of invariant sites (+I) and the gamma distribution for modeling rate heterogeneity (+G). The maximum likelihood trees were constructed using MEGA6 with Subtree-Pruning-Regrafting – Extensive (SPR level 5) as the LM Heuristic Methods. Each gene member assignment of common carp zinc transporter proteins was determined by phylogenetic analysis with zinc transporter proteins from zebrafish and human. Separate phylogenetic analyses were constructed per family using the same methodology with other representative vertebrate species including zebrafish, chicken, *Xenopus*, lizard, mouse and human. The ortholgoy analysis was conducted by analyzing synteny regions harboring SLC30A1 and SLC30A7. Shared synteny was searched by examining the conserved co-localization of neighboring genes on chromosome/scaffold of common carp and several vertebrates based on genome information from Ensembl.

### Expression of Zinc Transporter Genes

Total RNA from various adult common carp tissues (blood, brain, gill, heart, intestine, liver, muscle, skin, spleen, kidney) was extracted using Trizol reagent (Life technologies, NY, USA), and the cDNA was synthesized by the RT-PCR using the SuperScript III Synthesis System (Life technologies, NY, USA). ß-actin gene was used as an internal positive control, with forward primer (5′-TGCAAAGCCGGATTCGCTGG-3′) and reverse primer (5′-AGTTGGTGACAATACCGTGC-3′). The PCR comprised an initial denaturation step for 2 min at 94°C followed by 30 cycles of denaturation (30 sec at 94°C), annealing (30 sec at 60°C), and extension (20 sec at 72°C), and a final elongation step of 5 min at 72°C. The PCR products were separated by gel electrophoresis (1.5% agarose gel at 150 V) in the presence of ethidium bromide and visualized under ultraviolet light.

## Supporting Information

S1 Fig **Patterns of exon-intron architecture of SLC30 family.**
(TIF)

S2 Fig **Patterns of exon-intron architecture of SLC39 family.**
(TIF)

S1 Table **Gene names and accessions of reference SLC30 and SLC39 families used in this study.**
(DOCX)

## References

[1] Vallee BL, Falchuk KH (1993) The biochemical basis of zinc physiology. Physiol Rev 73:79–118. 8419966 10.1152/physrev.1993.73.1.79

[2] Fosmire GJ (1990) Zinc toxicity. Am J Clin Nutr 51:225–227. 2407097 10.1093/ajcn/51.2.225

[3] Zheng D, Feeney GP, Kille P, Hogstrand C (2008) Regulation of ZIP and ZnT zinc transporters in zebrafish gill: zinc repression of ZIP10 transcription by an intronic MRE cluster. Physiol Genomics 34:205–214. 18477665 10.1152/physiolgenomics.90206.2008 PMC2494845

[4] Huang L, Tepaamorndech S (2013) The SLC30 family of zinc transporters–A review of current understanding of their biological and pathophysiological roles. Mol Aspects Med 34:548–560. 23506888 10.1016/j.mam.2012.05.008

[5] Eide DJ (2004) The SLC39 family of metal ion transporters. Pflügers Archiv 447:796–800. 12748861 10.1007/s00424-003-1074-3

[6] Palmiter RD, Findley SD (1995) Cloning and functional characterization of a mammalian zinc transporter that confers resistance to zinc. The EMBO journal 14:639. 7882967 10.1002/j.1460-2075.1995.tb07042.x PMC398127

[7] Liuzzi JP, Cousins RJ (2004) Mammalian zinc transporters. Annu Rev Nutr 24:151–172. 15189117 10.1146/annurev.nutr.24.012003.132402

[8] Yan G, Zhang Y, Yu J, Yu Y, Zhang F, et al (2012) Slc39a7/zip7 plays a critical role in development and zinc homeostasis in zebrafish. PLoS One 7:e42939. 22912764 10.1371/journal.pone.0042939 PMC3418240

[9] Feeney GP, Zheng D, Kille P, Hogstrand C (2005) The phylogeny of teleost ZIP and ZnT zinc transporters and their tissue specific expression and response to zinc in zebrafish. Biochimica et Biophysica Acta (BBA)-Gene Structure and Expression 1732:88–95. 16500426 10.1016/j.bbaexp.2005.12.002

[10] Christoffels A, Bartfai R, Srinivasan H, Komen H, Orban L (2006) Comparative genomics in cyprinids: common carp ESTs help the annotation of the zebrafish genome. BMC Bioinformatics 7 Suppl 5: S2. 10.1186/1471-2105-7-S5-S2 PMC1764476 17254304

[11] Xu P, Li J, Li Y, Cui R, Wang J, et al Genomic insight into the common carp (*Cyprinus carpio*) genome by sequencing analysis of BAC-end sequences. BMC Genomics 12:188. 21492448 10.1186/1471-2164-12-188 PMC3083359

[12] Ji P, Liu G, Xu J, Wang X, Li J, et al (2012) Characterization of common carp transcriptome: sequencing, de novo assembly, annotation and comparative genomics. PLoS One 7:e35152. 22514716 10.1371/journal.pone.0035152 PMC3325976

[13] Jiang Y, Zhang S, Xu J, Feng J, Mahboob S, et al (2014) Comparative Transcriptome Analysis Reveals the Genetic Basis of Skin Color Variation in Common Carp. PLoS One 9:e108200. 25255374 10.1371/journal.pone.0108200 PMC4177847

[14] Xu J, Zhao Z, Zhang X, Zheng X, Li J, et al (2014) Development and evaluation of the first high-throughput SNP array for common carp (*Cyprinus carpio*). BMC Genomics 15:307. 24762296 10.1186/1471-2164-15-307 PMC4234440

[15] Xu P, Wang J, Wang J, Cui R, Li Y, et al (2011) Generation of the first BAC-based physical map of the common carp genome. BMC Genomics 12:537. 22044723 10.1186/1471-2164-12-537 PMC3221725

[16] Zhao L, Zhang Y, Ji P, Zhang X, Zhao Z, et al (2013) A dense genetic linkage map for common carp and its integration with a BAC-based physical map. PLoS One 8:e63928. 23704958 10.1371/journal.pone.0063928 PMC3660343

[17] Xu P, Zhang X, Wang X, Li J, Liu G, et al (2014) Genome sequence and genetic diversity of the common carp, *Cyprinus carpio* . Nat Genet. 10.1038/ng.3098 25240282

[18] Liu S, Li Q, Liu Z (2013) Genome-wide identification, characterization and phylogenetic analysis of 50 catfish ATP-binding cassette (ABC) transporter genes. PLoS One 8:e63895. 23696857 10.1371/journal.pone.0063895 PMC3655950

[19] Haney CJ, Grass G, Franke S, Rensing C (2005) New developments in the understanding of the cation diffusion facilitator family. J Ind Microbiol Biot 32:215–226. 10.1007/s10295-005-0224-3 15889311

[20] Jeong J, Eide DJ (2013) The SLC39 family of zinc transporters. Mol Aspects Med 34:612–619. 23506894 10.1016/j.mam.2012.05.011 PMC3602797

[21] Pocanschi CL, Ehsani S, Mehrabian M, Wille H, Reginold W, et al (2013) The ZIP5 ectodomain co-localizes with PrP and may acquire a PrP-like fold that assembles into a dimer. PLoS One 8:e72446. 24039764 10.1371/journal.pone.0072446 PMC3765157

[22] Ohno S (1970) Evolution by gene duplication: Springer-Verlag.

[23] Holland PW, Garcia-Fernàndez J, Williams NA, Sidow A (1994) Gene duplications and the origins of vertebrate development. Development 1994:125–133. 7579513

[24] Postlethwait JH, Yan Y-L, Gates MA, Horne S, Amores A, et al (1998) Vertebrate genome evolution and the zebrafish gene map. Nat Genet 18:345–349. 9537416 10.1038/ng0498-345

[25] McLysaght A, Hokamp K, Wolfe KH (2002) Extensive genomic duplication during early chordate evolution. Nat Genet 31:200–204. 12032567 10.1038/ng884

[26] Dehal P, Boore JL (2005) Two rounds of whole genome duplication in the ancestral vertebrate. PLoS Biol 3:e314. 16128622 10.1371/journal.pbio.0030314 PMC1197285

[27] Wang JT, Li JT, Zhang XF, Sun XW (2012) Transcriptome analysis reveals the time of the fourth round of genome duplication in common carp (*Cyprinus carpio*). BMC Genomics 13:96. 22424280 10.1186/1471-2164-13-96 PMC3352309

[28] David L, Blum S, Feldman MW, Lavi U, Hillel J (2003) Recent duplication of the common carp (*Cyprinus carpio L.*) genome as revealed by analyses of microsatellite loci. Mol Biol Evol 20:1425–1434. 12832638 10.1093/molbev/msg173

[29] Zhang X, Zhang Y, Zheng X, Kuang Y, Zhao Z, et al (2012) A consensus linkage map provides insights on genome character and evolution in common carp (*Cyprinus carpio L.*). Mar Biotechnol (NY) 15:275–312. 23073608 10.1007/s10126-012-9485-9

[30] Fraker PJ, King LE, Laakko T, Vollmer TL (2000) The dynamic link between the integrity of the immune system and zinc status. J Nutr 130:1399S–1406S. 10801951 10.1093/jn/130.5.1399S

[31] Hambidge M (2000) Human zinc deficiency. J Nutr 130:1344S–1349S. 10801941 10.1093/jn/130.5.1344S

[32] Liuzzi JP, Blanchard RK, Cousins RJ (2001) Differential regulation of zinc transporter 1, 2, and 4 mRNA expression by dietary zinc in rats. J Nutr 131:46–52. 11208937 10.1093/jn/131.1.46

[33] Glasauer SM, Neuhauss SC (2014) Whole-genome duplication in teleost fishes and its evolutionary consequences. Mol Genet Genomics: 1–16. 10.1007/s00438-014-0889-2 25092473

[34] Andrews GK, Wang H, Dey S, Palmiter RD (2004) Mouse zinc transporter 1 gene provides an essential function during early embryonic development. Genesis 40:74–81. 15452870 10.1002/gene.20067

[35] Chai F, Truong-Tran AQ, Ho LH, Zalewski PD (1999) Regulation of caspase activation and apoptosis by cellular zinc fluxes and zinc deprivation: a review. Immunol Cell Biol 77:272–278. 10361260 10.1046/j.1440-1711.1999.00825.x

[36] Lopez V, Kelleher SL (2009) Zinc transporter-2 (ZnT2) variants are localized to distinct subcellular compartments and functionally transport zinc. Biochem J 422:43–52. 19496757 10.1042/BJ20081189 PMC3381892

[37] Miletta MC, Bieri A, Kernland K, Schoni MH, Petkovic V, et al (2013) Transient Neonatal Zinc Deficiency Caused by a Heterozygous G87R Mutation in the Zinc Transporter ZnT-2 (SLC30A2) Gene in the Mother Highlighting the Importance of Zn (2+) for Normal Growth and Development. Int J Endocrinol 2013:259189. 24194756 10.1155/2013/259189 PMC3804372

[38] Seve M, Chimienti F, Devergnas S, Favier A (2004) In silico identification and expression of SLC30 family genes: an expressed sequence tag data mining strategy for the characterization of zinc transporters' tissue expression. BMC genomics 5:32. 15154973 10.1186/1471-2164-5-32 PMC428573

[39] Huang L, Gitschier J (1997) A novel gene involved in zinc transport is deficient in the lethal milk mouse. Nat Genet 17:292–297. 9354792 10.1038/ng1197-292

[40] Piletz J, Ganschow R (1978) Lethal milk mutation results in dietary zinc deficiency in nursing mice. Am J Clin Nutr 31:560–562. 637030 10.1093/ajcn/31.4.560

[41] Petkovic V, Miletta MC, Eble A, Fluck CE, Mullis PE (2014) Alteration of ZnT5-Mediated Zinc Import into the Early Secretory Pathway Affects the Secretion of Growth Hormone from Rat Pituitary Cells. Horm Res Paediatr 82:245–251. 25196974 10.1159/000365924

[42] Inoue K, Matsuda K, Itoh M, Kawaguchi H, Tomoike H, et al (2002) Osteopenia and male-specific sudden cardiac death in mice lacking a zinc transporter gene, Znt5. Hum Mol Genet 11:1775–1784. 12095919 10.1093/hmg/11.15.1775

[43] Huang L, Yu YY, Kirschke CP, Gertz ER, Lloyd KK (2007) Znt7 (Slc30a7)-deficient mice display reduced body zinc status and body fat accumulation. J Biol Chem 282:37053–37063. 17954933 10.1074/jbc.M706631200

[44] Huang L, Kirschke CP, Gitschier J (2002) Functional characterization of a novel mammalian zinc transporter, ZnT6. J Biol Chem 277:26389–26395. 11997387 10.1074/jbc.M200462200

[45] Yu YY, Kirschke CP, Huang L (2007) Immunohistochemical analysis of ZnT1, 4, 5, 6, and 7 in the mouse gastrointestinal tract. J Histochem Cytochem 55:223–234. 17101726 10.1369/jhc.6A7032.2006

[46] Palmiter RD, Huang L (2004) Efflux and compartmentalization of zinc by members of the SLC30 family of solute carriers. Pflügers Archiv 447:744–751. 12748859 10.1007/s00424-003-1070-7

[47] Gaither LA, Eide DJ (2000) Functional expression of the human hZIP2 zinc transporter. J Biol Chem 275:5560–5564. 10681536 10.1074/jbc.275.8.5560

[48] Gaither LA, Eide DJ (2001) The human ZIP1 transporter mediates zinc uptake in human K562 erythroleukemia cells. J Biol Chem 276:22258–22264. 11301334 10.1074/jbc.M101772200

[49] Kelleher SL, Lonnerdal B (2005) Zip3 plays a major role in zinc uptake into mammary epithelial cells and is regulated by prolactin. Am J Physiol Cell Physiol 288:C1042–1047. 15634741 10.1152/ajpcell.00471.2004

[50] Sun P, Wang S, Jiang Y, Tao Y, Tian Y, et al (2013) Zip1, Zip2, and Zip8 mRNA expressions were associated with growth hormone level during the growth hormone provocation test in children with short stature. Biol Trace Elem Res 155:11–22. 23921484 10.1007/s12011-013-9764-y

[51] Cousins RJ (2010) Gastrointestinal factors influencing zinc absorption and homeostasis. International Journal for Vitamin and Nutrition Research 80:243–248. 21462106 10.1024/0300-9831/a000030 PMC3777256

[52] Dufner-Beattie J, Kuo Y-M, Gitschier J, Andrews GK (2004) The adaptive response to dietary zinc in mice involves the differential cellular localization and zinc regulation of the zinc transporters ZIP4 and ZIP5. J Biol Chem 279:49082–49090. 15358787 10.1074/jbc.M409962200

[53] Geiser J, De Lisle RC, Andrews GK (2013) The zinc transporter Zip5 (Slc39a5) regulates intestinal zinc excretion and protects the pancreas against zinc toxicity. PLoS One 8:e82149. 24303081 10.1371/journal.pone.0082149 PMC3841122

[54] Kitamura H, Morikawa H, Kamon H, Iguchi M, Hojyo S, et al (2006) Toll-like receptor–mediated regulation of zinc homeostasis influences dendritic cell function. Nat immunol 7:971–977. 16892068 10.1038/ni1373

[55] Aydemir TB, Liuzzi JP, McClellan S, Cousins RJ (2009) Zinc transporter ZIP8 (SLC39A8) and zinc influence IFN-gamma expression in activated human T cells. J Leukoc Biol 86:337–348. 19401385 10.1189/jlb.1208759 PMC2726764

[56] Liuzzi JP, Lichten LA, Rivera S, Blanchard RK, Aydemir TB, et al (2005) Interleukin-6 regulates the zinc transporter Zip14 in liver and contributes to the hypozincemia of the acute-phase response. Proc Natl Acad Sci U S A 102:6843–6848. 15863613 10.1073/pnas.0502257102 PMC1100791

[57] Hojyo S, Miyai T, Fujishiro H, Kawamura M, Yasuda T, et al (2014) Zinc transporter SLC39A10/ZIP10 controls humoral immunity by modulating B-cell receptor signal strength. Proc Natl Acad Sci U S A 111:11786–11791. 25074919 10.1073/pnas.1323557111 PMC4136588

[58] Miyai T, Hojyo S, Ikawa T, Kawamura M, Irie T, et al (2014) Zinc transporter SLC39A10/ZIP10 facilitates antiapoptotic signaling during early B-cell development. Proc Natl Acad Sci U S A 111:11780–11785. 25074913 10.1073/pnas.1323549111 PMC4136617

