# Supplementary figures and images for: Genome Wide Identification, Phylogeny and Expression of Zinc Transporter Genes in Common Carp
Source: PLoS One. 2014 Dec 31;9(12):e116043. doi: 10.1371/journal.pone.0116043 (PMC4281218; doi:10.1371/journal.pone.0116043)

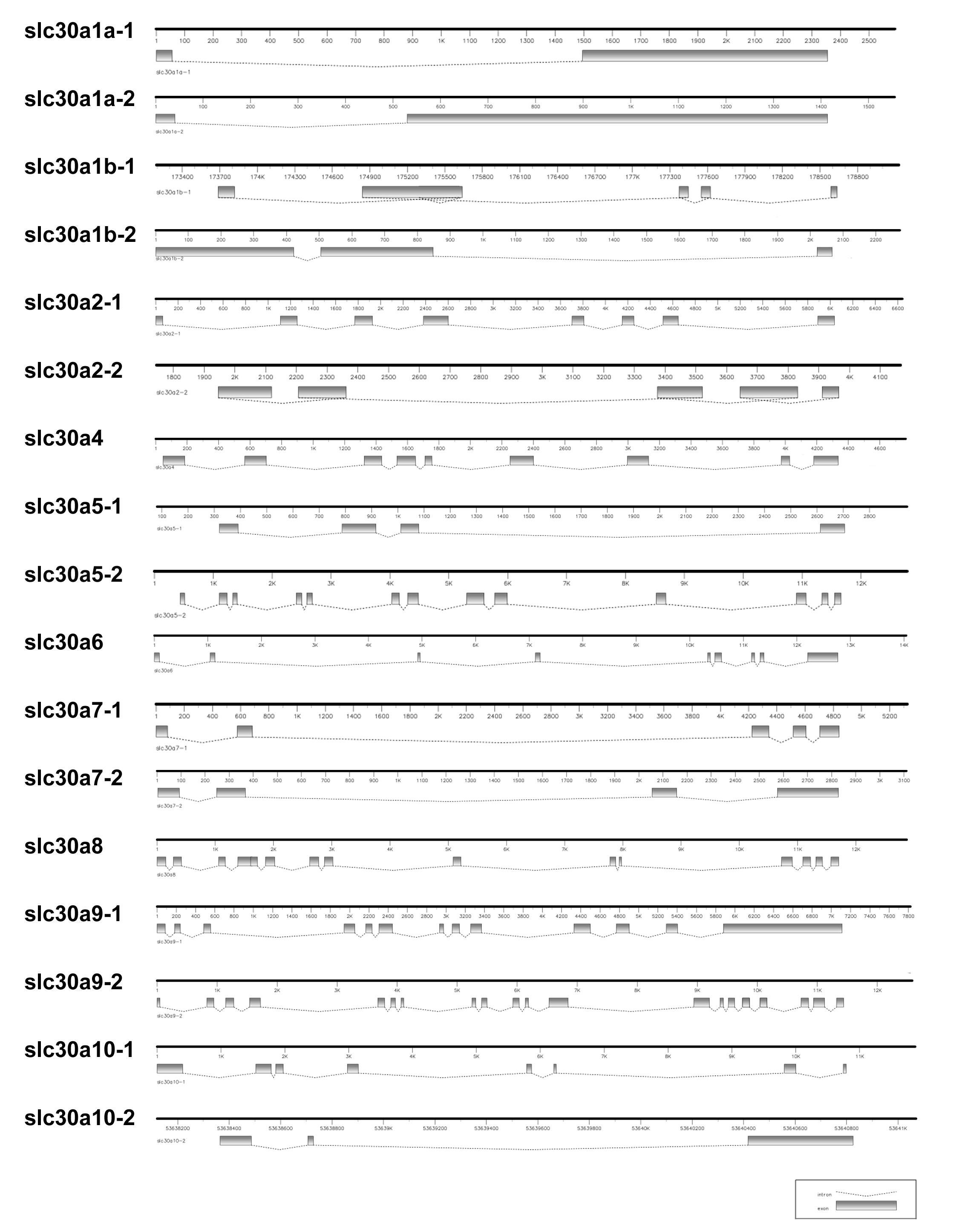

Supplement: S1 Fig — Patterns of exon-intron architecture of SLC30 family. (TIF) [file pone.0116043.s001.tif]

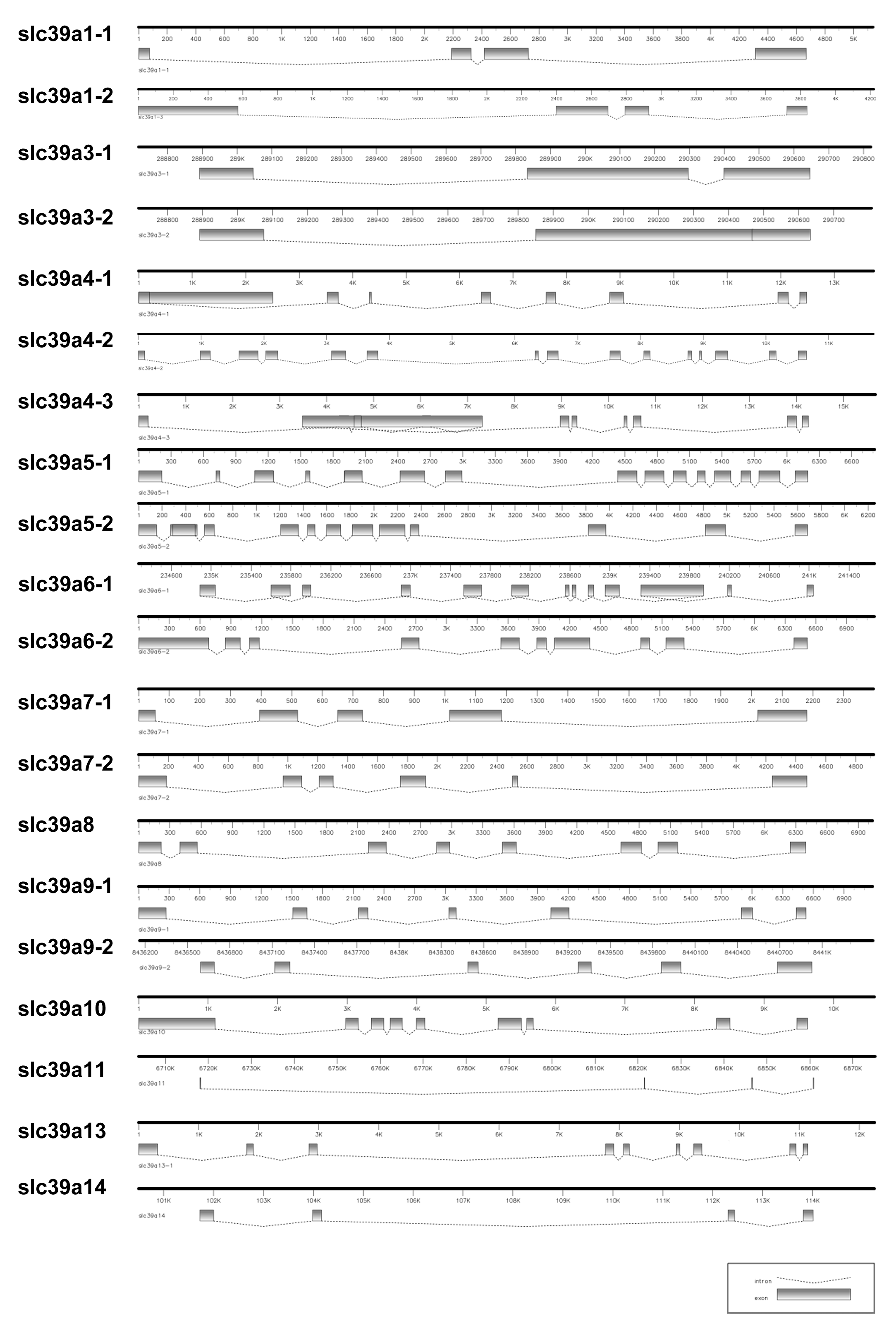

Supplement: S2 Fig — Patterns of exon-intron architecture of SLC39 family. (TIF) [file pone.0116043.s002.tif]
