# Supplementary material for: Genome Wide Identification, Phylogeny and Expression of Zinc Transporter Genes in Common Carp
Source: PLoS One. 2014 Dec 31;9(12):e116043. doi: 10.1371/journal.pone.0116043 (PMC4281218; doi:10.1371/journal.pone.0116043)
Supplement: S1 Table — Gene names and accessions of reference SLC30 and SLC39 families used in this study. (DOCX) [file pone.0116043.s003.docx]

Table S1. Gene names and accessions of reference SLC30 and SLC39 families used in this study.

| Gene | Zebrafish | Human | Mouse | Chicken | Lizard | Xenopus | Medaka |
| --- | --- | --- | --- | --- | --- | --- | --- |
| SLC30A1 | ENSDARG00000005463.5  ENSDARG00000053896.5 | ENSG00000170385.9 | ENSMUSG00000037434.7 | ENSGALG00000009858.4 | ENSACAG00000016353.3 | ENSXETG00000014427.3 | ENSORLG00000012247.1 |
| SLC30A2 | ENSDARG00000021305.10 | ENSG00000158014.11 | ENSMUSG00000028836.9 | ENSGALG00000010375.4 | ENSACAG00000017801.3 | ENSXETG00000009196.3 | ENSORLG00000008773.1 |
| SLC30A4 | ENSDARG00000007180.7 | ENSG00000104154.6 | ENSMUSG00000005802.7 | ENSGALG00000005703.4 | ENSACAG00000015702.1 | ENSXETG00000022040.3 | ENSORLG00000002464.1 |
| SLC30A5 | ENSDARG00000051921.3 | ENSG00000145740.15 | ENSMUSG00000021629.9 | ENSGALG00000014787.4 | ENSACAG00000016363.3 | ENSXETG00000000472.3 | ENSORLG00000013542.1 |
| SLC30A6 | ENSDARG00000077368.3 | ENSG00000152683.11 | ENSMUSG00000024069.7 | ENSGALG00000010618.3 | ENSACAG00000000973.2 | ENSXETG00000008384.3 | ENSORLG00000002108.1 |
| SLC30A7 | ENSDARG00000019998.7 | ENSG00000162695.8 | ENSMUSG00000054414.3 | ENSGALG00000020879.1 | ENSACAG00000003124.3 | ENSXETG00000025164.2 | ENSORLG00000014132.1 |
| SLC30A8 | ENSDARG00000057629.5 | ENSG00000164756.9 | ENSMUSG00000022315.3 | ENSGALG00000016118.4 | ENSACAG00000010841.3 | ENSXETG00000027945.2 | ENSORLG00000001769.1 |
| SLC30A9 | ENSDARG00000057272.5 | ENSG00000014824.10 | ENSMUSG00000029221.10 | ENSGALG00000014247.4 | ENSACAG00000012087.3 | ENSXETG00000021074.3 | ENSORLG00000000650.2 |
| SLC30A10 | ENSDARG00000034877.6 | ENSG00000196660.7 | ENSMUSG00000026614.6 | ENSGALG00000026727.1 | ENSACAG00000001203.2 | ENSXETG00000002721.3 |  |
|  |  |  |  |  |  |  |  |
| SLC39A1 | ENSDARG00000058257.2 | ENSG00000143570.14 | ENSMUSG00000052310.9 |  | ENSACAG00000010411.3 | ENSXETG00000019914.3 | ENSORLG00000002240.1 |
| SLC39A3 | ENSDARG00000016145.8 | ENSG00000141873.7 | ENSMUSG00000046822.7 | ENSGALG00000020582.4 |  | ENSXETG00000003715.2 | ENSORLG00000002333.1 |
| SLC39A4 | ENSDARG00000059361.5 | ENSG00000147804.6 | ENSMUSG00000063354.6 |  | ENSACAG00000027233.1 | ENSXETG00000016876.3 | ENSORLG00000018732.1 |
| SLC39A5 | ENSDARG00000079525.4 | ENSG00000139540.8 | ENSMUSG00000039878.7 | ENSGALG00000025854.1 | ENSACAG00000027233.1 | ENSXETG00000009499.3 |  |
| SLC39A6 | ENSDARG00000068143.4 | ENSG00000141424.9 | ENSMUSG00000024270.6 | ENSGALG00000013209.4 | ENSACAG00000001852.3 | ENSXETG00000014976.3 | ENSORLG00000006625.2 |
| SLC39A7 | ENSDARG00000036388.5 | ENSG00000112473.13 | ENSMUSG00000024327.10 |  |  | ENSXETG00000020367.3 | ENSORLG00000005862.1 |
| SLC39A8 | ENSDARG00000056757.3  ENSDARG00000087905.1 | ENSG00000138821.9 | ENSMUSG00000053897.10 | ENSGALG00000012298.4 | ENSACAG00000012798.3 | ENSXETG00000021329.3 | ENSORLG00000018167.1 |
| SLC39A9 | ENSDARG00000070447.4 | ENSG00000029364.8 | ENSMUSG00000048833.8 | ENSGALG00000009438.4 | ENSACAG00000005162.3 | ENSXETG00000017909.3 | ENSORLG00000009965.1 |
| SLC39A10 | ENSDARG00000005823.7 | ENSG00000196950.10 | ENSMUSG00000025986.6 | ENSGALG00000007777.4 | ENSACAG00000012301.3 | ENSXETG00000022134.3 | ENSORLG00000008760.1 |
| SLC39A11 | ENSDARG00000089127.1 | ENSG00000133195.8 | ENSMUSG00000041654.10 | ENSGALG00000004413.4 | ENSACAG00000008777.3 | ENSXETG00000009976.3 |  |
| SLC39A13 | ENSDARG00000000442.8 | ENSG00000165915.10 | ENSMUSG00000002105.10 | ENSGALG00000008122.2 | ENSACAG00000002440.3 | ENSXETG00000007914.3 | ENSORLG00000006101.1 |
| SLC39A14 | ENSDARG00000090174.1 | ENSG00000104635.10 | ENSMUSG00000022094.10 | ENSGALG00000028259.1 | ENSACAG00000010758.1 | ENSXETG00000010532.3 | ENSORLG00000019904.1 |
